# Replenishment of vitamin A for 7 days partially restored hepatic gene expressions altered by its deficiency in rats

**DOI:** 10.3389/fnut.2022.999323

**Published:** 2022-10-06

**Authors:** Yan Zhang, Kui Tian, Guoxun Chen

**Affiliations:** ^1^Department of Gastroenterology, Affiliated Puren Hospital of Wuhan University of Science and Technology, Wuhan, Hubei, China; ^2^Department of Radiology, Wuhan Pulmonary Hospital, Wuhan, Hubei, China; ^3^Department of Nutrition, University of Tennessee at Knoxville, Knoxville, TN, United States

**Keywords:** body weight, hepatic gene expression, plasma glucose, vitamin A deficient, vitamin A sufficient, Zucker rats

## Abstract

We investigated the effects of vitamin A (VA) status on metabolism of Zucker rats with different genders and genotypes, and of short-term refeeding of a VA sufficient (VAS) diet on VA deficient (VAD) animals. First, male and female Zucker lean (ZL) and fatty (ZF) rats at weaning were fed a VAD or VAS diet for 8 weeks. Second, male VAD ZL rats were fed a VAS diet for 3 (VAD-VAS3d) or 7 (VAD-VAS7d) days. The body weight (BW), blood parameters, and hepatic expressions of genes for metabolism were determined. VA deficiency reduced BW gain in ZL and ZF rats of either gender. VAD ZL rats had lower plasma glucose, insulin, and leptin levels than VAS ZL rats. VAD-VAS3d and VAD-VAS7d rats had higher plasma glucose, insulin, and leptin levels than that in the VAD rats. The hepatic mRNA levels of *Gck, Cyp26a1, Srebp-1c, Igf1, Rarb, Rxra, Rxrg, Pparg*, and *Ppard* were lowered by VA deficiency. Refeeding of the VAS diet for 3 days restored the *Gck* and *Cyp26a1* expressions, and for 7 days restored the *Gck, Cyp26a1, Igf1*, and *Rxrb* expressions significantly. The 7-day VA replenishment partially restored the hepatic gene expressions and metabolic changes in VAD ZL rats.

## Introduction

Chronic metabolic diseases have become a concern of global health. For example, global diabetes prevalence in 2019 was about 463 million, which number is anticipated to be 700 million in 2045 (1). This also increases the financial burden globally as the total diabetic expenditure was estimated to be 760 billion dollars in 2019 worldwide (2). On the other hand, the world is also facing a challenge to feed the ever-increasing population on earth. The global food production has to rise 50% by the year 2030 to meet this demand, which may create two scenarios, malnutrition in certain areas and overnutrition in others worldwide (3). Therefore, a clear understanding of nutrients’ roles will benefit human health globally.

As an essential and lipophilic micronutrient, vitamin A (VA, retinol) plays crucial roles in the general health of an individual, such as vision, growth, and immune responses (4, 5). The active metabolite of retinol, retinoic acid (RA), exists in multiple isomeric forms, such as all-*trans* RA and 9-*cis* RA. Retinol is reversibly oxidized into retinal by (retinol) alcohol dehydrogenases and short-chain dehydrogenases/reductases. The conversion of retinal to RA is an irreversible process catalyzed by (retinal) aldehyde dehydrogenases. RA regulates gene expression through the activations of two families of nuclear receptors, retinoic acid receptors (RARα, β, and γ), and retinoid X receptors (RXRα, β, and γ). Additional transcription factors such as hepatocyte nuclear factor 4α, and chicken ovalbumin upstream transcription factor II may also mediate RA’s effects on gene expressions (6, 7). RA is further catabolized into more polar metabolites for excretion by a RA-4-hydroxylase (gene *Cyp26a1*), a RA-responsive gene whose hepatic expression is induced after RA treatment (8). Recently, the effects of VA status on glucose and lipid metabolism have been recognized and appreciated (9, 10).

Metabolic abnormalities, such as obesity and diabetes, are often associated with profound changes in hepatic glucose and lipid metabolism (11, 12). In an attempt to find lipophilic molecules that influence insulin actions in hepatocytes, we found an activity that can modulate insulin-regulated expressions of the cytosolic form of phosphoenolpyruvate carboxyl kinase gene (*Pck1*) (13) and glucokinase gene (*Gck*) (14) in primary hepatocytes. The molecule responsible for this activity was identified as retinoids (14). RA also synergizes with insulin to induce the hepatic expression of sterol regulatory element-binding protein 1c gene (*Srebp-1c*), which is a master regulator of the expressions of genes responsible for the hepatic lipogenesis (15). The VA signaling regulates these genes’ expression *via* retinoic acid responsive elements (RAREs) on their promoters (6, 16, 17). All these demonstrate the role of VA signaling in the regulation of the expression levels of genes for the hepatic glucose and lipid metabolism.

Vitamin A status regulates body weight (BW), glucose, and lipid levels in animals. VA deficiency reduced BW gain (18) and depleted hepatic glycogen (19) in rats. On the other hand, excessive dietary VA intake in the form of retinyl esters significantly induced the hepatic glycogen content (20). The Zucker Fatty (ZF) rats are an animal model which carries a spontaneous mutation in the leptin receptor gene, causing obesity and insulin resistance. Zucker Diabetic Fatty (ZDF) rats are a well characterized animal model of type 2 diabetes (T2D). ZDF rats were derived by selectively inbreeding of ZF rats, which led to a sub-strain that develops overt T2D at around 10 weeks of age. Studies have shown that when male Zucker lean (ZL) and ZF rats were fed a VA sufficient (VAS) or VA deficient (VAD) diet for 8 weeks, the VAD diet prevented obesity and hyperlipidemia in ZF rats (21–24). VA status regulates the respiratory exchange ratio in ZL rats (24). Recently, we have shown that reduction of dietary VA status prevents the obesity and T2D in ZDF rats (25). ZF and ZDF rats have leptin receptor mutations that lead to hyperphagia, overnutrition, and development of obesity and T2D (26, 27). We have shown that the expression of retinaldehyde dehydrogenase 1 (*Raldh1*) gene level was elevated in the liver and hepatocytes of ZF rats (28), which might have led to the excessive hepatic RA production and increase in lipogenesis. The *Raldh1* knockout mice are resistant to diet-induced obesity and insulin resistance (29). This was attributed to that the knockout mice have elevated retinaldehyde levels in adipose tissues, which probably suppressed adipogenesis (29). In humans with T2D, the plasma VA levels, and VA intake show some variations depending on population studied (30–33). Interestingly, RA treatment was shown to benefit blood glucose control in ZDF rats (34), and *ob/ob* mice (35). All these demonstrate that VA and its signaling play a role in the regulation of metabolism.

Given the important roles of VA in the regulation of metabolism, we hypothesize that the replenishment of VA in the diet will restore the expression levels of hepatic genes for glucose and lipid metabolism in the VAD animals. Here, ZL and ZF rats were fed a VAD diet for 8 weeks, and then ZL rats were refed a VAS diet for 3 or 7 days. We report that refeeding of a VAS diet for 3 or 7 days partially restored the hepatic expression levels of genes altered by VA deficiency in ZL rats.

## Materials and methods

### Reagents

The reagents for collecting plasma glucose were obtained from DiaSys Diagnostic Systems GmbH (Germany). The ELISA kits for insulin (#ml302840), leptin (#ml002969), and glucagon (#ml600102) were purchased from Shanghai Enzyme-linked Biotechnology Company (China). Reagents for cDNA synthesis, real-time PCR and other compounds were obtained from Shanghai Sangon Biotech Company (China).

### Animals and diets

Male ZL (*fa*/+ or +/+) and ZF (*fa/fa*) rats were bred and maintained in the animal facility of the Taikang Medical Diagnosis Service (Hebei) Ltd. (Guan, Hebei, China). They were housed in colony cages in a temperature and humidity-controlled environment. The synthetic VAD and VAS diets were isocaloric and contained 4.05 kcal/g (1 cal = 4.184 J) diet, which had been used previously (23). The synthetic basal diet contained 18.3, 22.1, and 59.6% energy from protein, fat (ether extract), and carbohydrate, respectively. Both diets had 10% (w/w) fat, in which the contents of total saturated, monounsaturated, and polyunsaturated fatty acids were 2.72, 3.31, and 3.42%, respectively. The VAS diet contained 22.1 IU VA/g diet, whereas the VAD diet contained 0 IU VA/g diet.

Two sets of experiments were conducted to evaluate the impacts of VA status on BW gain and metabolism in ZL and ZF rats. The first one was aimed to determine whether Zucker rats with different genotypes (+/+, +/*fa*, and *fa/fa*) and genders (male and female) respond differently to a VAD diet. Both male and female ZL (+/+ or *fa*/+) or ZF rats after weaning (3 weeks of age) were fed a VAD or a VAS diet for 8 weeks. Their BW and body length were measured weekly. The second experiment was aimed to determine whether refeeding of a VAS diet for 3 or 7 days will promote BW gain and restore the expression levels of hepatic genes involved in glucose and lipid metabolism. Here, ZL rats (+/+ and *fa*/+) after weaning were fed a VAD or VAS diet for 8 weeks. At the end of the 8-week feeding, the VAD rats were fed the VAS diet for 3 or 7 additional days. The 8-week protocol was sufficient to induce VA deficiency according to the original observation by McCollum and Davis (1913) (18) and our previous studies (21, 23). All animals were cared for in accordance with the Guide to the Care and Use of Experimental Animals (36). All procedures were approved by the Institutional Animal Care and Use Committee of Wuhan Puren Hospital (No.: 2020-10).

### Genotyping

The genotypes of ZL (+/+ and *fa*/+) and ZF (*fa/fa*) rats were determined using a PCR based method as described in Durham et al. (37). In brief, a piece of ear tissue was obtained *via* punching, added to 75 μl of alkaline lysis buffer (25 mM NaOH/0.2 mM EDTA), and incubated at 95°C for 30 min to release genomic DNA. The lysate was mixed with 75 μl of 40 mM Tris–HCl buffer for neutralization and spun at 20,000 × *g* for 5 min to obtain supernatant, which was used as the template for PCR amplification. Each PCR reaction with a 25 μl volume contained 2.5 μl tissue lysate as the template, 2.5 μl 10 × PCR buffer, 2.5 μl 25 mM MgCl2, 1 μl 100 mM dNTP, 0.5 μl 5U/μl Taq DNA polymerase, 1 μl each of 10 μm forward (5′-CGTATGGAAGTCACAGA-3′) and reverse (5′GAATTCTCTAAATATTTCAGC-3′) primers, and 14 μl water. The reverse primer contains a single base substitution (C to G) at the 3′ end, which generates a *Pvu*II site in the wild type allele only. The PCR conditions were: 95°C for 5 min, and 40 cycles of 95°C for 30 s, 60°C for 30 s, and 72°C for 30 s. After that, 20 μl of the PCR product were digested in a 28 μl reaction containing 10 units of *Pvu*II at 37°C for 1 h, and subjected to gel electrophoresis in a 4% agarose gel with 10 mg/mL ethidium bromide and 0.5 × Tris-borate-EDTA buffer. The wildtype genotype (+/+), heterozygous (*fa*/+), and homozygous (*fa/fa*) genotypes showed the presence of one 80 bp band, 80 and 101 bp bands, and one 101 bp band, respectively.

### Plasma and tissue sample collection and measurements

At the end of feeding, animals were fasted for 6 h after the diets were removed at around 7:00 AM, and euthanized under CO2 before tissue and plasma sample collections. Accumulated venous blood was collected into EDTA coated tubes and centrifugated at 2,000 ×*g* and 4°C for 20 min to obtain the plasma. The liver of both male and female rats, and two pads of epididymal white adipose tissue (WAT) of male rats was removed, weighted, and immediately frozen in liquid nitrogen. Tissue and plasma samples were stored at −80°C until be further processed. The plasma glucose level was determined using kits from DiaSys Diagnostic Systems GmbH (Germany) following the manufacture’s manual. The plasma insulin, leptin and glucagon levels were measured using the ELISA kits according to the manufacture’s manuals.

### RNA extraction and real-time PCR

To extract total RNA, about 100 mg frozen rat liver sample was added to 1 ml of TRIzol reagent (#15596026, Thermo Fisher, Shanghai, China) according to the manufacture’s protocol. Any contaminated DNA was removed using a DNA-free kit, and 2 μg of DNA-free RNA was used for the synthesis of cDNA. Each SYBR green based real-time PCR reaction in have a volume of 14 μl and contained cDNA reverse-transcribed from 14 ng of total RNA, 2.33 pmol forward and reverse primers, and 7 μl of 2 × SYBR Green PCR Master Mix (Applied Biosystems). The gene specific primers that will be provided upon request were used to amplify the corresponding cDNA, and some of them have been used previously (13, 14). PCR reactions in triplicates were carried out in 96-well plates using a 7300 Real-Time PCR system and were conducted as follow: 50°C for 2 min, 95°C for 10 min, followed by 40 cycles of 95°C for 15 s and 60°C for 1 min. The expression level of a particular gene was shown as -△ cycle threshold (CT) value by subtracting the CT value of 36B4 (an invariable control gene) from the CT value of the indicated gene.

### Statistics analysis

SPSS 23.0 software was used for statistical analysis. Student’s *t*-test with 95% confidence interval and one-way ANOVA with least significance difference *post-hoc* analysis tests were performed to compare two and more than two groups, respectively. If needed, natural log transformation was performed before analysis. Data were presented as means ± S.E.M. The *p*-value less than 0.05 was considered significantly different.

## Results

### Vitamin A deficiency reduced body weight gain and body fat in Zucker lean and Zucker fatty male rats

The effects of VA deficiency on ZL (+/+ or *fa*/+) or ZF (*fa/fa*) male rats were analyzed after they had been fed a VAD or a VAS diet for 8 weeks. As shown in Table 1, the initial BW of ZL male rats at weaning (3 weeks of age) fed a VAD diet was similar to that of the VAS group. At the end of the 8-week dietary treatment, VAD ZL male rats had significantly lower BW, body length, and BW gain than those of VAS rats (*P* < 0.05). In ZF male rats, the parameters of BW, length, and BW gain after 8 weeks followed the same trend as those in ZL rats (*P* < 0.05), demonstrating the reduced somatic growth when the rats were fed a VAD diet (*P* < 0.05). The liver weight, but not the liver/BW ratio, of the ZL male VAD rats was significantly lower than that of VAS rats. However, the liver/BW ratios in the ZF male rats were significantly different between VAD and VAS groups (*P* < 0.05). The WAT weight and the WAT/BW ratio of VAD male rats, regardless of lean or fatty, were all significantly lower than those of VAS rats (*P* < 0.05). All these data demonstrated that VA deficiency reduces the BW, liver weight and fat pad weight in male Zucker rats. There appears to be no difference between wild type (+/+) and heterozygous (+/*fa*) ZL male rats fed the VAD or VAS diet.

**TABLE 1:** Body weight (BW), length, liver weight, white adipose tissue (WAT) weight, liver/BW ratio, WAT/BW ratio, and BW gain in male+/+, *fa*/+, and *fa/fa* Zucker rats fed a vitamin A deficient (VAD) or vitamin A sufficient (VAS) diet for 8 weeks.

Genotype	Diets (*n*)	Start BW (g)	End BW (g)	BW gain (g)	Length (cm)	Liver (g)	WAT (g)	Liver/BW	WAT/BW
+/+	VAD (7)	36.0 ± 4.2	170.8 ± 33.6a	134.8 ± 32.0a	34.9 ± 2.6	5.7 ± 0.7	0.70 ± 0.3a	0.034 ± 0.008a/b	0.004 ± 0.002a/b
	VAS (5)	32.9 ± 5.6	294.1 ± 29.5a′,*	261.3 ± 24.8a′,*	38.2 ± 1.4*	8.9 ± 0.9a′,*	3.10 ± 0.9a′,*	0.030 ± 0.003a′	0.010 ± 0.003a′, *
*fa*/+	VAD (8)	37.1 ± 4.7	166.1 ± 32.7a	129.0 ± 29.1a	34.9 ± 3.4	5.7 ± 0.6	0.59 ± 0.4 a	0.035 ± 0.006b	0.003 ± 0.002a
	VAS (8)	35.8 ± 5.6	254.5 ± 45.5a′’*	218.7 ± 46.9a′,*	37.3 ± 2.2	8.7 ± 1.3a′,*	2.51 ± 0.8a′,*	0.035 ± 0.009b′	0.010 ± 0.002a′, *
*fa/fa*	VAD (7)	36.5 ± 2.3	244.9 ± 33.6b	208.5 ± 33.6 b	34.6 ± 1.59	6.6 ± 1.7	3.86 ± 3.8 b	0.027 ± 0.006a	0.014 ± 0.013b
	VAS (8)	37.2 ± 5.6	445.4 ± 38.6b′*	408.1 ± 38.3b′*	37.2 ± 5.6*	17.2 ± 1.8b′,*	15.1 ± 1.24b′,*	0.039 ± 0.003b′,*	0.034 ± 0.003b′, *

BW, body weight; cm, centimeters; g, grams; VAD, vitamin A deficient; VAS, vitamin A sufficient; WAT, epididymal white adipose tissue; n, animal number in the indicated group. *For comparing VAD with VAS in the same genotype using student’s t-test; a < b, a′ < b′ for comparing the different genotypes within the same diet using one way ANOVA; All *P* < 0.05.

### Vitamin A deficiency reduced body weight gain and body fat in Zucker lean and Zucker fatty female rats

To evaluate whether female Zucker rats respond to the change of VA status, ZL and ZF female rats were fed a VAD or VAS diet for 8 weeks. Table 2 shows their BW, body length and liver weight, and liver/BW ratio of ZL (+/+ and *fa*/+) and ZF (*fa/fa*) female rats. Since female rats do not have epididymal fat, we did not have fat data. Different from that of the ZL male rats, values of body length of ZL female rats were similar between VAD and VAS groups. The difference in body length between VAD and VAS groups was only observed in ZF female rats (*P* < 0.05). The liver weight in VAD female rats, but not the liver/BW ratio, was still significantly lower than their corresponding VAS female rats in the same genotype. As in the ZL male rats, no difference was observed between wild type (+/+) and heterozygous (*fa*/+) ZL female rats fed the VAD or VAS diet.

**TABLE 2:** Body weight (BW), body length, liver weight, liver/BW ratio, and BW gain in female +/+, *fa*/+, and *fa/fa* Zucker rats fed a vitamin A deficient (VAD) or vitamin A sufficient (VAS) diet for 8 weeks.

Genotype	Diets (*n*)	Start BW (g)	End BW (g)	BW gain (g)	Length (cm)	Liver (g)	Liver/BW
+/+	VAD (8)	33.0 ± 2.9	128.8 ± 21.3a	93.8 ± 20.7a	32.4 ± 3.1	4.7 ± 0.6	0.038 ± 0.008
	VAS (5)	34.3 ± 6.7	180.1 ± 18.3a′, *	145.8 ± 17.9a′, *	33.9 ± 1.5	6.1 ± 0.9a′, *	0.034 ± 0.003a′
*fa*/+	VAD (8)	33.7 ± 3.3	139.9 ± 26.8a	106.3 ± 25.8a	32.4 ± 2.3	4.8 ± 0.5	0.035 ± 0.006
	VAS (8)	35.4 ± 4.5	175.1 ± 19.0a′, *	139.7 ± 17.3a′, *	32.9 ± 3.2	6.9 ± 0.4a′, *	0.040 ± 0.005b′, *
*fa/fa*	VAD (8)	36 ± 2.8	238.3 ± 66.4b	202.3 ± 63.8b	31.3 ± 2.9	7.0 ± 0.8	0.031 ± 0.007
	VAS (7)	31.0 ± 3.4*	336.8 ± 60.8b′, *	305.8 ± 59.4b′, *	34.6 ± 2.0*	11.4 ± 1.5b′, *	0.034 ± 0.004a′

BW, body weight; cm, centimeters; g, grams; VAD, vitamin A deficient; VAS, vitamin A sufficient; n, animal number in the indicated group. *For comparing VAD with VAS in the same genotype using student’s t-test; a < b, a′ < b′ for comparing the different genotypes within the same diet using one way ANOVA; All *P* < 0.05.

As for female rats fed the VAD or VAS diet, end BW and BW gain in ZL (+/+, *fa*/+) rats were obviously lower than that in ZF (*fa/fa*) rats, presenting similar trend as male ones (*P* < 0.05). The liver weight of ZF female rats fed the VAS diet was higher (11.4 g) than that of +/+ and *fa*/+ ZL rats (6.1 and 6.9 g, respectively). Interestingly, the live/BW ratio of heterozygous ZL (*fa*/+) female rats (0.040) was higher than that of ZF (*fa/fa*) female rats (0.034). All results indicate for the first time that ZL (+/+ and *fa*/+) and ZF female rats responded to the VAD diet largely like those male ones.

### Refeeding of a vitamin A sufficient diet for 3 or 7 days increased body weight in Zucker lean male rats

**FIGURE 1:**
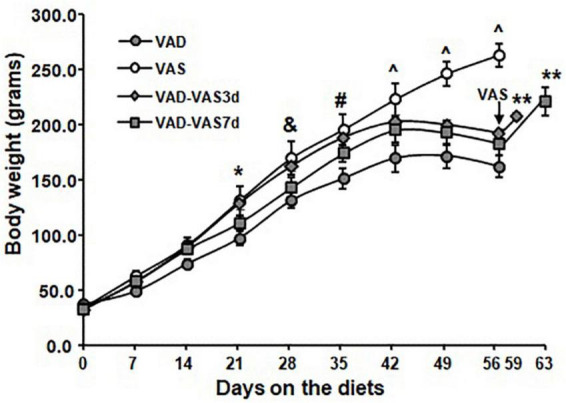
The body weight (BW) of Zucker lean (ZL) rats fed a vitamin A sufficient (VAS), a vitamin A deficient (VAD) diet for 8 weeks, or those fed a VAD diet for 8 weeks and followed by the VAS diet for 3 (VAD-VAS3d) or 7 (VAD-VAS7d). Mean ± SEM, *n* = 8 for each group; * For VAD < VAS, & for VAD/VAD-VAS7d < VAD-VAS3d/VAS; # for VAD and VAD-VAS7d < VAS and VAD < VAD-VAS3d, ^VAD < VAD-VAS3d < VAS and VAD-VAS7d < VAS using one way ANOVA; ** for comparing VAD-VAS3d or VAD-VAS7d at the indicated date with the BW of the group at 56 days (8 weeks).

As stated in our previous results, the responses to VAD or VAS are largely similar between male and female rats. Therefore, in order to determine the effects of refeeding of a VAS diet on the BW regain, ZL (+/+ and *fa*/+) male rats after weaning (3 weeks old) only were selected and then fed a VAS or a VAD diet for 8 weeks. After that, the VAD rats were refed the VAS diet for 3 or 7 days. As shown in Figure 1, ZL male rats fed a VAD diet grew at a similar rate as those fed a VAS diet did for the first 2 weeks. Starting from the week 3 (21 days), ZL male rats fed a VAD diet gained significantly less BW than those fed a VAS diet did (*P* < 0.05). After on the diets for 4 weeks (28 days), the BW values of rats in the VAD and VAD-VAS7d groups were lower than that of VAD-VAS3d and VAS groups (&). After on the diets for 5 weeks (35 days), the BW values of rats in the VAD and VAD-VAS7d groups were lower than that in the VAS group, and that in the VAD group were lower than that in the VAD-VAS3d group (#). After on the diets for 6 (42 days) to 8 weeks (56 days), the BW values of rats in the VAS group were higher than that in the VAD-VAS3d and VAD-VAS7d groups, and that in the VAD group were lower than that in VAD-VAS3d group (^). The BW values of ZL rats fed the VAD diet peaked at the sixth week, and started to drop after that. The BW of ZL rats fed the VAS diet continued to rise throughout the 8-week study period (*P* < 0.05). The BW of the ZL rats in the VAD-VAS-3d group were higher than that of VAD and VAD-VAS-7d group at week 4 (*P* < 0.05). When the VAS diet was refed to the VAD rats, the ZL rats in VAD-VAS3d and VAD-VAS7d groups started to gain significant amount of BW at 3 (59 days) and 7 (63 days) days comparing with that at the end of the 8-week (56 days) feeding of the VAD diet (^**^). These results demonstrate that restoration of VA in the diet promoted BW gain in ZL rats.

### Refeeding of a vitamin A sufficient diet for 3 or 7 days increased plasma glucose, insulin, and leptin levels

Plasma glucose, insulin, leptin, and glucagon levels were measured in ZL rats fed a VAS or a VAD diet for 8 weeks, or those fed a VAD diet and followed by the VAS diet for 3 (VAD-VAS3d) or 7 (VAD-VAS7d). Table 3 shows that VAD ZL rats had lower plasma glucose, insulin, and leptin levels than the VAS ZL rats. The refeeding of the VAS diet for 3 or 7 days respectively increased the plasma levels of glucose, insulin, and leptin in ZL rats of the VAD-VAS3d and VAD-VAS7d groups significantly in comparison with that of the VAD group (*P* < 0.05). There was a trend of reduction of plasma glucagon levels in rats of the VAD-VAS3d and VAD-VAS7d groups in comparison with that of VAD and VAS groups, but did not reach statistical significance, probably due to large variations of the data. The increases in plasma insulin, leptin and glucose levels demonstrate that VA sufficiency promotes anabolism.

**TABLE 3:** Plasma glucose, insulin, leptin, and glucagon in Zucker lean (ZL) rats fed a vitamin A sufficient (VAS) or a vitamin A deficient (VAD) diet for 8 weeks, or those fed a VAD diet and followed by the VAS diet for 3 (VAD-VAS3d) or 7 (VAD-VAS7d).

Diets	Glucose (mg/dL)	Insulin (ng/mL)	Leptin (ng/mL)	Glucagon (pg/mL)
VAD	70.88 ± 9.06*	0.30 ± 0.08*	0.61 ± 0.23*	121.61 ± 77.09
VAS	100.25 ± 5.60	1.57 ± 0.75a	2.48 ± 2.47	171.08 ± 210.67
VAD + VAS 3 days	102.43 ± 9.38	0.68 ± 0.20	1.39 ± 0.94	67.47 ± 19.02
VAD + VAS 7 days	97.71 ± 8.28	0.66 ± 0.16	1.36 ± 0.57	65.43 ± 19.21

Plasma glucose, insulin, leptin, and glucagon were measured in ZL rats fed a VAS or a VAD diet for 8 weeks, or those fed a VAD diet and followed by the VAS diet for 3 (VAD-VAS3d) or 7 (VAD-VAS7d). Mean ± SEM, n = 8 for each group; *P < 0.05, as compared with VAS, VAD-VAS 3 days and VAD-VAS 7 days; a *P* < 0.05, for comparing with VAD-VAS 3 days, VAD-VAS 7 days.

### Refeeding of a vitamin A sufficient diet for 3 or 7 days partially recovered the hepatic expression levels of genes involved in vitamin A, glucose, and lipid metabolism

**FIGURE 2:**
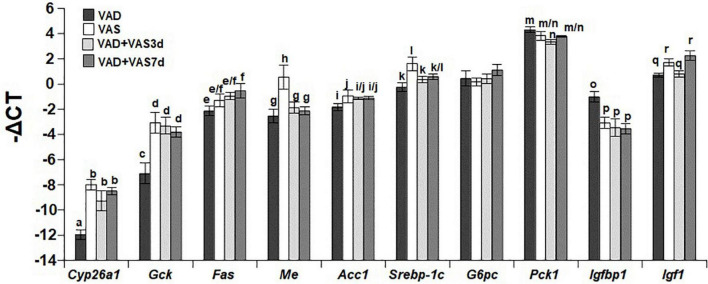
The hepatic mRNA levels of *Cyp26a1, Gck, Fas, Me, Acc1, Srebp-1c, G6pc, Pck1, Igfbp1*, and *Igf1* in Zucker lean (ZL) rats fed the vitamin A deficient (VAD) for 8 weeks, the vitamin A sufficient (VAS) diet for 8 weeks, or fed the VAD diet for 8 weeks followed by the VAS diet for 3 (VAD + VAS3d) or 7 (VAD + VAS 7d) days. Data are presented as mean ± SEM of minus delta Ct (cycle threshold); *n* = 7, 5, 5, and 5 for the VAD, VAS, VAD-VAS3d, and VAD-VAS7d, respectively; a < b for *Cyp26a1* (cytochrome P450 26a1), c < d for *Gck* (glucokinase), e < f for *Fas* (fatty acid synthase), g/h for *Me* (malic enzyme), i < j for *Acc1* (acetyl CoA carboxylase alpha), k < l for *Srebp-1c* (sterol regulatory element-binding protein 1c), m > n for *Pck1* (cytosolic form of phosphoenolpyruvate carboxykinase), o > p for *Igfbp1* (insulin like growth factor-binding protein 1), q < r for *Igf1* (insulin-like growth factor 1); all *P* < 0.05.

We next compared the hepatic mRNA levels of *Cyp26a1* (cytochrome P450 26a1 gene as an indicator of VA status), *Gck* (glucokinase gene in glycolysis), *Fas* (fatty acid synthase gene in the lipogenic pathway), *Me* (malic enzyme gene for the production of NADPH), *Acc1* (acetyl-CoA carboxylase gene in the lipogenic pathway), *Srebp-1c* (a key gene in lipogenesis), *G6pc* (Glucose-6-phosphatase catalytic subunit gene in the gluconeogenic pathway), *Pck1* (phosphoenolpyruvate carboxykinase gene in the gluconeogenic pathway), *Igfbp1* (Insulin-like growth factor-binding protein-1 gene for the binding of insulin-like growth factor 1), and *Igf1* Insulin-like growth factor 1 gene for the regulation of growth and metabolism) in ZL male rats of the VAD, VAS, VAD + VAS3d, and VAD + VAS7d groups. As shown in Figure 2, the hepatic expression level of *Cyp26a1* in ZL rats of the VAD group was significantly lower than that of VAS, VAD-VAS3d, and VAD-VAS7d, showing the improvement of VA status after refeeding of the VAS diet for 3 and 7 days in VAD-VAS3d and VAD-VAS7d groups. The expression level of *Gck* mRNA followed the same trend.

The hepatic expression level of *Fas* mRNA in the VAD-VAS7d, but not that in the VAS and VAD-VAS3d groups, was higher than that in the VAD group, suggesting the increase in the hepatic fatty acid synthesis after feeding the VAS diet for 7 days. Interestingly, the hepatic levels of *Me* mRNA in the VAS group was significantly higher than that VAD, VAD-VAS3d, and VAD-VAS7d groups, which are not different among them. The hepatic levels of *Acc1* and *Srebp-1c* mRNA in the VAS group were higher than that in the VAD group, which was not changed after refeeding of the VAS diet in the VAD-VAS3d and VAD-VAS-7d groups.

The expression levels of *G6pc* mRNA were not different among the four groups. The *Pck1* mRNA level in the VAD group was higher than that in the VAD-VAS3d group, but not different from that in the VAS and VAD-VAS7d groups. The *Igfbp1* mRNA level in the VAD group was significantly higher than that in the VAS, VAD-VAS3d, and VAD-VAS7d groups, showing the impacts of the VA status on metabolism. The *Igf1* mRNA levels in the VAD and VAD-VAS3d groups were lower than that in the VAS and VAD-VAS7d groups, showing that it took 7 days to restore the *Igf1* expression in the liver.

### Refeeding of a vitamin A sufficient diet for 3 or 7 days partially recovered the hepatic expression levels of nuclear receptors responsible for vitamin A signaling

**FIGURE 3:**
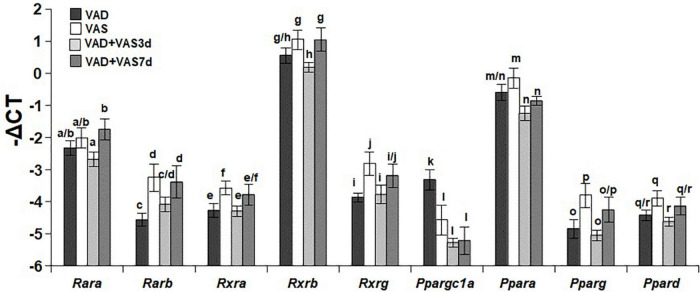
The hepatic mRNA levels of *Rara, Rarb, Rxra, Rxrb, Rxrg, Ppargc1a, Ppara, Pparg*, and *Ppard* in Zucker lean (ZL) rats fed a vitamin A deficient (VAD) or vitamin A sufficient (VAS) diet for 8 weeks or fed a VAD diet for 8 weeks and fed a VAS diet for 3 (VAD + VAS 3d) or 7 (VAD + VAS 7d) days. Data are presented as mean ± SEM of minus delta Ct (cycle threshold); *n* = 7, 5, 5, and 5 for the VAD, VAS, VAD-VAS3d, and VAD-VAS7d, respectively; a < b for *Rara* (retinoic acid receptor alpha), c < d for *Rarb* (Retinoic acid receptor beta), e < f for *Rxra* (rexinoid X receptor alpha), g > h for *Rxrb* (rexinoid X receptor beta), i < j for *Rxrg* (rexinoid X receptor gamma), k > l for *Ppargc1a* (peroxisome proliferative activated receptor gamma coactivator 1 alpha), m > n for *Ppara* (peroxisome proliferative activated receptor alpha), o < p for *Pparg* (peroxisome proliferative activated receptor gamma), q > r for *Ppard* (peroxisome proliferative activated receptor delta or beta).

To determine whether the restoration of VA status affects the expression levels of nuclear receptors involved in mediating RA signaling and regulating glucose and fatty acid metabolism, we compared the hepatic mRNA levels of *Rara* (retinoic acid receptor alpha gene), *Rarb* (retinoic acid beta gene), *Rxra* (retinoid X receptor alpha gene), *Rxrb* (retinoid X receptor beta gene), *Rxrg* (retinoid X receptor gamma gene), *Ppargc1a* (peroxisome proliferator-activated receptor gamma coactivator 1 alpha gene), *Ppara* (peroxisome proliferator-activated receptor alpha gene), *Pparg* (peroxisome proliferator-activated receptor gamma gene), and *Ppard* (peroxisome proliferator-activated receptor delta gene), of the ZL rats in the four dietary groups. As shown in Figure 3, the hepatic levels of *Rara* mRNA in the VAD and VAS groups were not different, whereas that in the VAD-VAS7d group was higher than that in the VAD-VAS3d group. The level of *Rarb* mRNA in the VAS group was higher than that in the VAD group, which was restored by the feeding of the VAS diet for 7, but not 3 days (c < d). The mRNA levels of *Rxra, Rxrg, Pparg* and *Ppard* in the VAS group were higher than that in the VAD and VAD-VAS3d groups, whereas that in the VAD-VAS7d group had a trend to return, but did not reach statistical significance. The mRNA levels of *Rxrb* in the VAS and VAD-VAS7d groups were higher than that in the VAD-VAS3d group. The mRNA level of *Ppargc1a* in the VAD group was higher than that in VAS, VAD-VAS3d, and VAD-VAS7d groups. The mRNA level of *Ppara* in the VAS group was higher than that in the VAD-VAS3d and VAD-VAS7d groups.

## Discussion

Here, we evaluated the effects of VA status on the BW gain in wild type (+/+), heterozygous (*fa*/+), and homozygous (*fa/fa*) Zucker rats of both genders, and the expression levels of hepatic genes in response to the refeeding of a VAS diet for 3 and 7 days in ZL male rats. We observed that VA deficiency leads to less BW gain in +/+, *fa*/+, and *fa/fa* rats of both genders. In addition, the refeeding of a VAS diet to VAD ZL male rats partially restored the expression levels of hepatic genes involved in metabolic controls.

We have shown previously that ZL and ZF male rats fed a VAD diet have lower BW than those fed a VAS diet for 8 weeks (21–24). This is the first time that the responses of female ZL (+/+ and *fa*/+) and ZF rats to a VAD diet were presented. Generally, the responses of female Zucker rats to the VAD diet like those of the male ones. The responses of BW and liver weight of +/+ and *fa*/+ female rats to VA deficiency do not differ from each other in either VAD or VAS condition, indicating the equivalence of the wild type and heterozygous ZL rats regarding the VA status.

Therefore, male ZL rats with both +/+ and *fa*/+ genotypes were used in the VAS refeeding experiment after they were weaned (3 weeks of age). Male ZL rats were fed the VAD diet for 8 weeks, and then those VAD rats were fed a VAS diet for 3 or 7 days. The refeeding of a VAS diet for 3 or 7 days is sufficient to increase the BW of those VAD rats significantly. What is noteworthy, we also noticed that ZL rats assigned to the VAD-VAS-3d had higher BW than the VAD and VAD-VAS-7d groups at certain time points, showing their slightly differential responses to the VAD diet during the course to develop VA deficiency. We attributed this difference to the variations of the original VA storage in ZL rats at weaning as they were derived from different breeding pairs, mothers with slightly different nutritional status and age. It is difficult to obtain animals with the same VA status at weaning, which is why we designed an 8-week protocol to ensure the animals in VAD groups to reach VA deficiency. Nevertheless, the BW gain of ZL rats in all three VAD groups stopped at round 6 weeks, demonstrating the success of VA deficiency. This served our original purpose to induce VA deficiency before the replenishment. The BW gain started to increase when the dietary VA became available, demonstrating clearly that VA plays an anabolic role in rats.

We have shown previously that the flux of dietary VA induces the expression of hepatic genes responsible for lipogenesis, such as *Gck* and *Srebp-1c* in the VAD ZL rats fed a VAS, but not a VAD diet (24). This occurs 6 h later after the intake of a VAS diet in the VAD ZL rats (24). Interestingly, in the current experimental setting, the expression levels of *Gck*, but not *Srebp-1c*, *Me* and *Acc1*, mRNA levels in the ZL VAD rats fed the VAS diet for 3 or 7 days were returned to that of the VAS rats. The *Fas* mRNA level in VAD-VAS7d, but not VAD-VAS3d, group is significantly higher than that in the VAD group. All these indicate that the presence of VA for 3 or 7 days in those VAD rats increase certain, but not all, mRNA levels of genes whose expression levels have been altered due to the VAD status.

Here, we demonstrate that the feeding of a VAS diet to the VAD rats restores their hepatic gene expression profile toward that of the VAS rats differentially. For certain genes such as *Cyp26a1, Gck, ppargc1a*, and *Igfbp1*, a 3-day refeeding of a VAS diet is sufficient, whereas for others such as *Rarb* and *Rxrb*, it took 7 days. As far as we know, this is the first time that the expression levels of genes involved in the hepatic glucose, and lipid metabolism are measured after VA returns to the body. Regarding the transcription factors and cofactors, the changes of *Ppargc1a* is similar to that of *Gck* and *Igfbp1*. On the other hand, the expression levels of *Rarb* and *Rxrb* in the VAD-VAS7d, but not VAD-VAS3d, group returned to level of the VAS group, which is similar to that of *Igf1*. It has been known that the binding of IGFBP-1 to IGF1 could reduce the IGF-1’s action in metabolism during fasting (38). Our data show that the hepatic mRNA levels of *Igfbp-1* and *Igf1* are regulated differentially by the VA status. Actually, the hepatic *Igfbp-1* mRNA expression is regulated by insulin (39). The hepatic *Igfbp-1* mRNA is independent of circulating IGF-1 in humans. It is reasonable as there are other IGFBPs (40).

All these results show that the mRNA levels of those genes in the VAD rats return to the levels of VAS rats at different paces. For some of them, refeeding of a VAS diet for 3 days is sufficient. However, for others, the refeeding of a VAS for 7 days might not even enough. Whether this phenomenon is caused by the change of VA metabolism after the return of VA in the body or due to any permanent change of the gene expression due to VA deficiency remains to be answered. Future experiments with longer refeeding of a VAS diet are needed to answer this question.

We have also shown that VA status regulates the respiratory exchange ratio in ZL rats (24). It will be interesting to see how many days is sufficient for the VAD rats after refed a VAS diet to increase the anabolism as those VAS rats. Whether returning to a normal anabolism precedes the gene expression changes or after the recovery of the gene expression is an excellent future project.

Our current project has some limitations. First, we did not include groups with longer refeeding time of the VAS diet to show whether the expression levels of those genes changed by VA deficiency really can recover or not. This can be done in the future. Second, we only tested the refeeding in ZL rats, but not ZF rats. It will be interesting to see whether the reduced BW or the correction of obesity in VAD ZF rats is a transient phenomenon due to VA deficiency or a permanent change. Third, we only measured the hepatic gene expression due to limited resources. It will be interesting to see whether the gene expression levels also change in other tissues such as fat. This can be done in the future as well.

## Conclusion

In summary, both female and male ZL and ZF rats fed a VAD diet have lower BW gain, and liver mass than that fed a VAS diet, clearly demonstrating the role of VA in anabolism. Refeeding of a VAS diet in VAD ZL rats is sufficient to restore BW gain and partial expressions of genes affected by VA deficiency. These data demonstrated that the VAD rats are excellent molecular nutrition models to study the effects of VA on glucose and lipid metabolism after reintroducing VA in the animal body. They will certainly help our understanding of gene regulation *in vivo* and underlying mechanisms by which overnutrition leads to the development of metabolic diseases such as obesity and T2D.

## Data availability statement

The original contributions presented in this study are included in the article/supplementary material, further inquiries can be directed to the corresponding author.

## Ethics statement

This animal study was reviewed and approved by Institutional Animal Care and Use Committee of Wuhan Puren Hospital (No.: 2020-10).

## Author contributions

YZ and KT contributed equally to this study as co-first authors. KT contributed to manuscript writing and data analyses. YZ and GC contributed to design the study and final revision of the manuscript. All authors have read and approved the submission.
